# Control and Manipulation of Nano Cracks Mimicking Optical Wave

**DOI:** 10.1038/srep17292

**Published:** 2015-11-27

**Authors:** Young D. Suh, Junyeob Yeo, Habeom Lee, Sukjoon Hong, Jinhyeong Kwon, Kyunkyu Kim, Seung Hwan Ko

**Affiliations:** 1 Applied Nano and Thermal Science Lab, Department of Mechanical Engineering, Seoul National University, 1 Gwanak-ro, Gwanak-gu, Seoul 151-742, Korea (R.O.K); 2 Department of Mechanical Engineering, University of California, Berkeley, CA 94720-1740, USA

## Abstract

Generally, a fracture is considered as an uncontrollable thus useless phenomenon due to its highly random nature. The aim of this study is to investigate highly ordered cracks such as oscillatory cracks and to manipulate via elaborate control of mechanical properties of the cracking medium including thickness, geometry, and elastic mismatch. Specific thin film with micro-sized notches was fabricated on a silicon based substrate in order to controllably generate self-propagating cracks in large area. Interestingly, various nano-cracks behaved similar to optical wave including refraction, total internal reflection and evanescent wave. This novel phenomena of controlled cracking was used to fabricate sophisticated nano/micro patterns in large area which cannot be obtained even with conventional nanofabrication methods. We also have showed that the cracks are directly implementable into a nano/micro-channel application since the cracks naturally have a form of channel-like shape.

There is wide interest in development of nano-patterning techniques based on a silicon substrate intended for interdisciplinary studies including nano-fluidics^1^, nano-electronics^2^ and nanoelectromechanical systems (NEMS)^3^. Various nano-patterning techniques utilizing lithography as well as non-lithographic approaches are available nowadays^4^. Several frequently used and nanoscale precision assured techniques include electron beam lithography (EBL), scanning probe microscopy (SPM) or scanning tunneling microscopy (STM), and atomic force microscopy (AFM)^5^,^6^,^7^,^8^. The resolution of these techniques are highly precise, however the fabrication processes are often time consuming and expensive. In addition, serial processes are required for large area patterns because a single process area of these techniques is usually smaller than 1 cm × 1 cm. On the other hand, nanoimprinting, self-assembly, and block-copolymer lithography techniques have attained patterning capabilities equivalent or even better than in some respect to photolithography at relatively lower cost^9^,^10^,^11^,^12^. These techniques are typically carried out under non-vacuum and room temperature environment which leads to reduced cost and processing steps. Nevertheless, there are limitations of these techniques: resultant patterns are simple repetitive types, and fabrication of a master template which often involves the aid of state of art optical lithography technology is required. Foremost, both techniques are hindered by the difficulties of defect free fabrication over large area. For these reasons, large area nanopatterning is a challenging assignment.

Since discovery of cracking control in glass material by Yuse and Sano^13^, various research have introduced possibility of the cracking as a patterning method and have found applications especially in micro and nanofluidics^14^,^15^ and electronics^16^,^17^. In turn, awareness of using cracking as a viable candidate for nanopatterning technique have steadily emerged from being a totally random phenomenon^15^,^18^,^19^,^20^,^21^,^22^. Recently, the prospect of controlled nanocrack as a nanopatterning method has been revitalized by Koo *et al*.^23^ Using chemical vapor deposition (CVD) of glassy material on a silicon substrate, precise control of crack initiation and stop of different propagation modes of fracture such as straight and oscillatory crack have been successfully demonstrated. The cracks in Koo’s report showed great nanoscale features including extremely good line edge roughness (LER), line width roughness (LWR), and aspect ratio of width to depth. Another similar attempt has been made by Kim *et al*.^24^ by utilizing differential thermal expansion of glassy films on a metal patterned silicon substrate. Although there have been some instabilities in the oscillation of the crack, control of wave parameters of the oscillatory crack has been successfully demonstrated in microscale. Those studies showed remarkable control of naturally propagating oscillatory cracks as a mean of nanopatterning. In an attempt to expand this prospective phenomenon even further for various applications including nano/micro channel fabrication, we demonstrate elaborate control of the oscillatory cracks including initiation, stop, and manipulation of wave properties at a specific location or within a region of interest while maintaining high aspect ratio, low LER, and LWR of the cracks, using CVD deposition of glassy films on a silicon. In addition, we find that the oscillatory cracks fabricated in this manner exhibit optical wave-like behaviors such as refraction, total internal reflection and evanescent wave. Use of these unique optical wave like characteristics of the oscillatory crack enables various nanopatterns which are directly applicable to nanochannel fabrication.

## Results and Discussions

**Figure 1:**
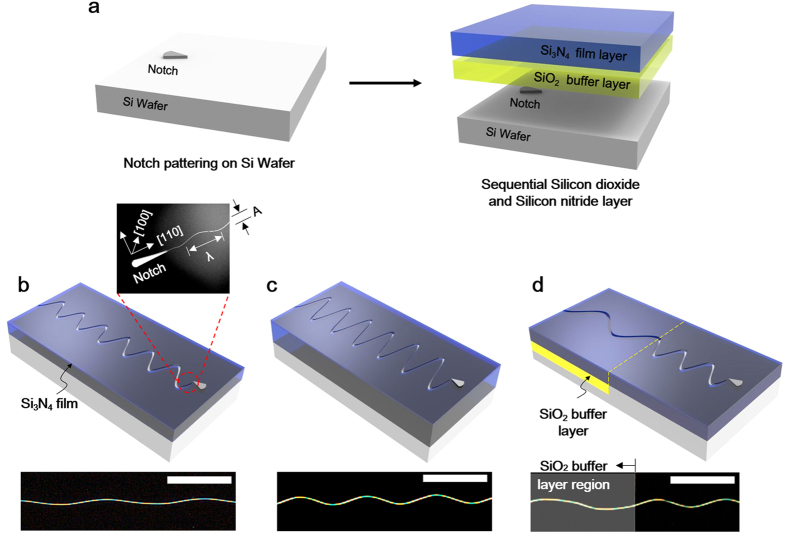
Schematic diagram of crack generation and control. (**a**) Structure used for nanocrack generation. (**b–d**) An oscillatory crack generated on Si_3_N_4_ film with propagation direction in [110] on (100) silicon wafer and its corresponding optical microscopic image. Inset scale is 100 μm. Notice that the wavelength and amplitude of the oscillatory crack can be manipulated (**c**) by changing Si_3_N_4_ layer thickness and by (**d**) introducing SiO_2_ buffer layer.

In order to generate an oscillatory crack, a cracking medium composed of Si_3_N_4_ or Si_3_N_α_ and SiO_2_ is deposited on a Si substrate as illustrated in Fig. 1a. In such system, residual stress after deposition process induces tensile stress in the thin film^25^. When the thin film is deposited on a notch structure, either positive or negative pattern shape, an acute stress concentration is induced at the notch tip. The tip also provides an initial flaw for a fracture to grow upon. As a result, a channeling crack is initiated to release the stored elastic energy in the Si_3_N_4_ thin film at the tip as shown in Fig. 1b. The cracks initiated in this fashion self-propagate in steady oscillatory manner in [110] direction on (100) silicon substrate until the point where another stress concentration that surmounts the driving force is reached^26^. Although the exact cause of the oscillation is not clearly defined yet, it appears to be related to the crystal structure of underlying silicon substrate. Under identical experimental condition, the oscillatory cracks have not been discovered on (111) oriented Si substrates. On (110) oriented Si substrates, oscillatory cracks have been observed, however straight cracks predominantly occurs unlike the (100) Si substrate case. (See supplementary information Fig. S2) The simplest method for manipulation of the oscillatory cracks in Si_3_N_4_/Si film-substrate system is to regulate Si_3_N_4_ deposition thickness which influences the amplitude of oscillatory cracks (Fig. 1c). Figure 1b,c shows increased Si_3_N_4_ film thickness results in substantial amplitude increase of the oscillatory cracks. Simultaneously, the wavelength of the oscillatory crack varies with the film thickness. In order to manipulate the wavelength of the oscillatory crack separately, a SiO_2_ buffer layer is introduced between Si substrate and Si_3_N_4_ thin film. As shown in Fig. 1d, the wavelength of oscillatory crack is considerably increased by the buffer layer with negligible amplitude difference.

**Figure 2:**
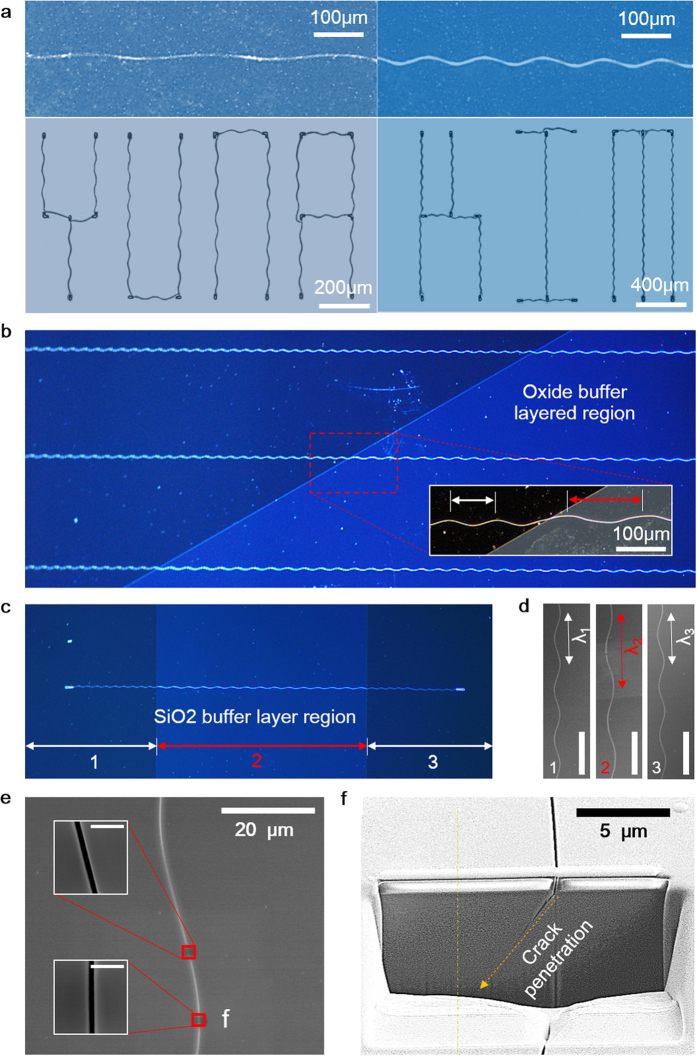
Wave property control of sinusoidal nanocrack. (**a**) (top) Oscillatory cracks with different amplitude and wavelength using different Si_3_N_4_ film deposition thickness and underlying buffer oxide layer. (bottom) Letters (“**YUNA KIM**”) drawn by crack control. (**b**) Wavelength modulation with oxide buffer layer. Note the high aspect ratio nanopatterns each of which having nanoscale width and centimeter scale length. (**c,d**) An oscillatory crack is initiated and stopped with modulated wavelength at the center with buffer layer. Note that the wavelength of the oscillatory cracks are reversible when it passes through different regions. The wavelength is significantly increased in the silicon dioxide buffer layer region, and corresponding SEM images of the region 1, 2, and 3 are shown. The inset scale bar is100 μm. (**e**) Width of a nanocrack in a typical oscillatory crack. The inset scale bar is 1 μm. (**f**) Cross sectional image of an oscillatory crack in depth direction obtained via FIB. Note that the tip of cross-sectional crack profile was not vertical but it points to the center line of in-plane propagation direction of the oscillatory crack.

Complementary introduction of the oxide buffer layer between the Si_3_N_4_ film and Si wafer enables manipulation of both wavelength and amplitude of the oscillatory crack. Top left image of Fig. 2a shows an oscillatory crack generated on 900 nm Si_3_N_4_ film with 50 nm SiO_2_ buffer layer. The wavelength is ~200 μm and the amplitude is roughly 1/20 of the wavelength. On the contrary, the wavelength of oscillatory cracks found on 1.2 μm Si_3_N_4_ film without oxide buffer layer is ~100 μm and the amplitude is roughly 1/10 of the wavelength as shown in Fig. 2a (top right). Therefore, combinatory use of specific Si_3_N_4_ film thickness that corresponds to target amplitude and the SiO_2_ buffer layer provides a utility for wavelength and the amplitude control. Combined with crack notch and stop design, various arbitrary patterns such as letters (*YUNA KIM*) with various oscillatory crack wavelength were demonstrated (Fig. 2a).

Selectively etched SiO_2_ patterns prior to Si_3_N_4_ film deposition enables localized control of wave characteristics of the oscillatory crack as shown in Fig. 2b,c. When an oscillatory crack propagates over the SiO_2_ buffer layer region along in-plane direction, the wavelength of the oscillatory crack increases at the interface of two regions while amplitude remains unchanged. In Fig. 2b, multiple parallel oscillatory cracks are generated by placing a notch array in parallel manner. Inset of Fig. 2b confirms noticeably increased wavelength of an oscillatory crack on the oxide buffer layer. Unless there are special constraints such as defects or other structures including crack stop or notches, the cracks propagate continuously in the buffer layer region in the same way as they do in non-buffer region. The wavelength of the oscillatory crack is dependent on the underlying layer. As shown in Fig. 2c, an oscillatory crack initiated from a notch has initially shorter wavelength (region “**‘1**”). Once the crack enters the oxide buffer layers (region “**2**”),the wavelength becomes elongated. When it leaves the oxide buffered layer region and reaches non-buffer layer region (region “**3**”), the increased wavelength comes back to its initial wavelength. This wavelength shifting is totally reversible regardless of the shape of underlying buffer layer. Corresponding SEM images of the oscillatory crack in each regions confirm locally increased wavelength of the oscillatory crack in the buffer layer region, shown in Fig. 2d. The oxide buffer layer has little effect on width of the crack channel. Figure 2e shows the width of a typical oscillatory crack which is approximately 120 ~ 150 nm. Although there are slight variations depending on the location or geometry of structures near the cracks, the width of the oscillatory cracks in oxide buffer region is not considerably different from that of non-buffer region. In addition, the width of the oscillatory crack has no direct relationship with other wave parameters such as wavelength and amplitude (see supplementary information Fig. S3).

In any cases of the oscillatory cracks, significant substrate crack penetration through the depth direction was observed. This penetration of the crack into the Si substrate is always much deeper than the thickness of the Si_3_N_4_ thin film due to high elastic energy of the Si_3_N_4_ film^27^. It was revealed that the tip of cross-sectional crack profile was not vertical but it points to the center line of in-plane propagation direction of the oscillatory crack. The maximum penetration angle occurs at the peaks of the wave and the corresponding angle coincides with (111) plane of the Si (100) substrate as revealed in FIB (focused ion beam) cross section image shown in the Fig. 2e. As the oscillatory crack completes one cycle of sine wave, the penetration angle rotates from approximately 55˚ to −55˚ degrees with respect to center of axis which coincides with in-plane propagation direction of the oscillatory crack. This substrate penetration is closely related to the cause of oscillatory cracks. The surface energy of crystal silicon’s cleavage plane varies with number of broken bonds. The number ratio is known to be 1:(3/2)^(1/2)^:3^(1/2)^ for {111}, {110}, and {100} respectively for Si (100)^28^. The alteration of penetration angle between one {111} plane to another {111} requires fracture in higher surface energy plane inevitably, thus the oscillatory cracks are preferred under higher stress state than the straight cracks assuming that there are no defects.

**Figure 3:**
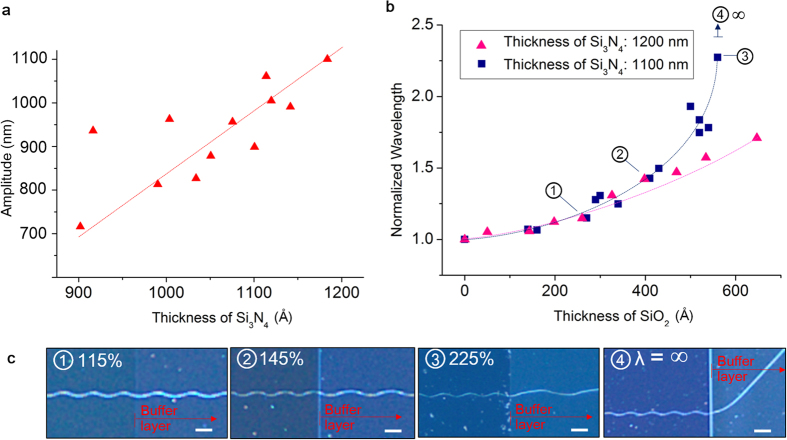
Wavelength and amplitude control of sinusoidal nanocrack. (**a**) Normalized wavelength and amplitude of the oscillatory cracks plotted against different Si_3_N_4_ deposition thicknesses. (**b**) Normalized wavelength of the oscillatory cracks plotted against SiO_2_ buffer layer thickness and (**c**) corresponding optical microscope images of oscillatory cracks. Scale bar is100 μm.

In a typical thin film/substrate system with an assumption that the film and substrate are elastically homogeneous and the substrate is substantially thicker than the film, the energy release rate for initiation,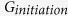 and steady state propagation of a channeling crack, 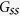 are


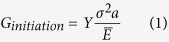



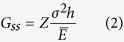


where 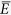, 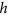, and 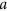 are plane strain tensile modulus, thickness of the thin film, and initial flaw size, respectively and 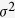, Y and 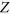 are uniform tensile loading due to residual stress, dimensionless quantity for crack initiation and steady state propagation, respectively^27^,^29^. In the Si_3_N_4_/Si film-substrate system, crack initiation rate of the oscillatory crack significantly increases as the film thickness 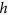 increases since the thicker Si_3_N_4_ film thickness results in higher strain energy in the system^27^. All cracks including random and oscillatory form are rarely found when 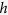 is less than 900 nm because insufficient amount of residual stress for the initiation is established whereas initiation rate is greatly improved where 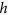 is increased to near 1.1 μm. From those results, it is conjectured that increased Si_3_N_4_ film thickness leads to higher residual tension 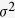 in the given system. Similar to crack initiation rate, amplitude of oscillatory crack is related to Si_3_N_4_ film thickness. As shown in Fig. 3a, the amplitude of the oscillatory crack has a proportional relationship with the Si_3_N_4_ film thickness although there are some uncertainties due to unintended structures such as defects.

In order to independently control the wavelength from the amplitude in the oscillatory crack and to have the film thickness fixed at optimized value for highest oscillatory crack initiation, additional thin film layer is employed under Si_3_N_4_ film. SiO_2_ is an ideal candidate due to its compatibility with micro fabrication process and no significant total film thickness increase. The SiO_2_ buffer layer promotes favorable effect on the given system with relatively small increase in the overall thickness of the Si_3_N_4_/SiO_2_ multi-layered thin film. The range of this additional layer thickness required for wavelength manipulation is up to 1000 Å which is relatively small as compared to 1.1 μm of Si_3_N_4_ film thickness at optimal initiation rate. As shown in Fig. 3b, the SiO_2_ buffer layer thickness under 700 Å is sufficient to convey wavelength increase up to 225% while the amplitude change in the oscillatory crack, which is primarily governed by the film thickness, is restrained. The wavelength of the oscillatory cracks has exponential relationship with the buffer layer thickness as shown in Fig. 3b. When the SiO_2_ buffer layer thickness is larger than 700 Å for 1.2 μm of Si_3_N_4_ film, oscillatory crack propagation mode dramatically changes into straight mode. When the Si_3_N_4_ film thickness is reduced, wavelength increase rate per unit SiO_2_ buffer layer thickness is also increased. The result shown in Fig. 3b,c indicates that the wavelength of the oscillatory crack increase rate surges with reduced Si_3_N_4_ film thickness.

**Figure 4:**
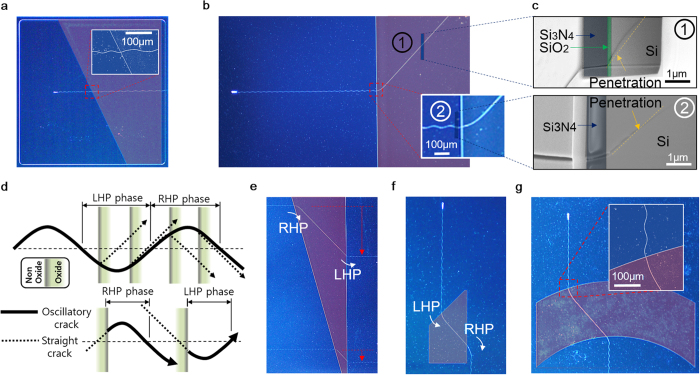
Optical wave-like behavior of nanocracks and their direction control. (**a**) Wavelength modulation when it pass through thin SiO_2_ buffer layer. (**b**) Propagation mode changed from oscillatory mode to straight mode by thick SiO_2_ buffer layer and (**c**) cross sectional (FIB) image of straight (top) and oscillatory (bottom) crack. (**d**) Selection of crack kinking direction when oscillatory crack impinging into SiO_2_ buffer layer region (top) and when straight crack exits the SiO_2_ buffer oxide region (bottom). (**e**) Propagation phase shifted by straight crack travel distance. (**f**) Propagation phase shifted leftward direction using SiO_2_ buffer layer. (**g**) Propagation direction manipulated by SiO_2_ buffer layer patterned like an optical lens.

Interestingly, the oscillatory crack passing through different buffer layer regions shows a unique behavior similar to that of light. Complementary use of various SiO_2_ buffer layer patterns and wavelength manipulation enables fabrication of sophisticated nano-patterns. As shown in Fig. 4a, wavelength of an incident oscillatory crack wave in the SiO_2_ buffer layer region becomes elongated as if the wavelength of an optical wave increases when it propagates through a medium with lower refractive index. Bending of an oscillatory crack’s propagation direction was also observed just like the refraction optical wave. As shown in Fig. 4b, the propagation direction and mode were changed when passing through SiO_2_ buffer layer. The buffer layer with thickness greater than 70 nm turns the crack’s propagation mode from oscillatory to straight. At in-plane interface of SiO_2_ buffer layer, the incident oscillatory crack kinks to conform to straight crack’s principle propagation direction. As a result, the in-plane propagation direction is changed from [110] to [100], which are the principle propagation directions of the each modes on (100) Si substrate, respectively. When the straight crack leaves the buffer oxide region, exactly opposite phenomenon occurs. As shown in the cross section image (Fig. 4c) attained using FIB, the straight crack in the SiO_2_ region (upper image) has crack penetration angle similar to that of oscillatory crack at the incident phase (lower image). This indicates the incident crack penetration angle is maintained throughout the propagation in the SiO_2_ buffer layer region, thus the phase of incident wave at the interface determines the in-plane kinking direction.

At an arbitrary phase of the oscillatory crack, corresponding penetration angle with respect to the principle propagation axis of the oscillatory crack always lies between −55˚ and 0˚ or +55˚ and 0˚. For simplicity, positive phase which is right hand side with respect to the principle propagation axis of the oscillatory crack from the view point towards crack tip is defined as right hand phase (RHP) and the opposite is defined as left hand phase (LHP). When the phase is in RHP range, the penetration angle is also in the positive range. In such case, the crack kinks to the left at the interface, and vice versa as shown in Fig. 4d. Similarly, when the crack exits the buffer layer region, the straight crack turns into the oscillatory crack. The straight crack’s penetration angle does not exceed its maximum angle of 55˚, and the penetration angle of the incident phase is maintained, therefore when the straight crack exits the buffer region, it kinks to the opposite direction of the initial kinking direction at the incident where transition occurred from oscillatory to straight mode. In order to successfully control kinking direction, wavelength control is crucial in terms of anticipating the exact path of the propagation and of realizing elaborate crack patterning.

Using these optical wave-like characteristics of the oscillatory crack with elaborate control of incident phase and angle at in-plane interface of SiO_2_ buffer layer, precise manipulation of nano crack can be realized, including propagation shift. Figure 4e shows selective shift of the two waves by adjusting propagation length of straight crack portion. The propagation shift in opposite direction can also be attained as shown in Fig. 4f. When the incident angle between the oscillatory wave and the interface is properly controlled, the cracks can be focused into a specific location as if the optical wave is focused by lens as shown in Fig. 4g.

**Figure 5:**
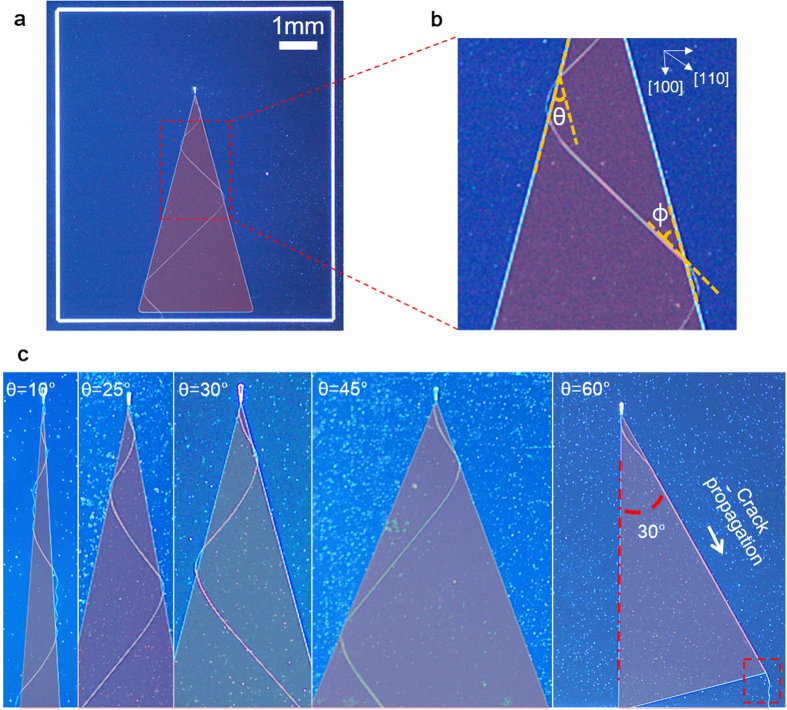
Optical wave-like behavior of oscillatory nanocracks and their amplitude amplification. (**a,b**) Optical image of amplified oscillatory crack and its magnified view. (**c**) Optical images of ascending amplification of the wave of a sinusoidal nanocrack for various apex angles of the buffer SiO_2_ pattern and the crack propagated along the interface.

Other optical wave-like characteristics of the oscillatory crack include amplification, total internal reflection, and evanescent wave. Using these unique optical wave like characteristics of the oscillatory crack enables sophisticated manipulation of nano-cracks for the fabrication of arbitrary nano-patterns over large area. By placing a triangular shape SiO_2_ buffer layer with an apex facing a notch tip, an initiated crack meanders two adjacent sides of the triangle due to refraction at the edges of the triangle as if total internal reflection (TIR) of optical wave (Fig. 5a). In terms of amplitude, this can be seen as amplification. The degree of amplification is governed by the angle of apex, *θ*. As shown in Fig. 5b, the angle *ф*, between the interface and the crack tip determines the final propagation direction of the crack. When *ф* is sufficiently large (>15˚), the crack kinks back into the buffer layer region and starts propagating to the opposite direction. This process is repeated, in turn unique and sophisticated nano-patterns are fabricated, as shown in Fig. 5a. When the crack tip angle coincide with the boundary of the SiO_2_ buffer layer region where *ф* is <15˚, the crack propagates along the edge of the buffer layer. Despite the principle propagation direction of the straight crack is [110] on (100) Si wafer, the straight crack propagates along the edge as shown in Fig. 5c. This phenomenon resembles evanescent wave of optical wave.

**Figure 6:**
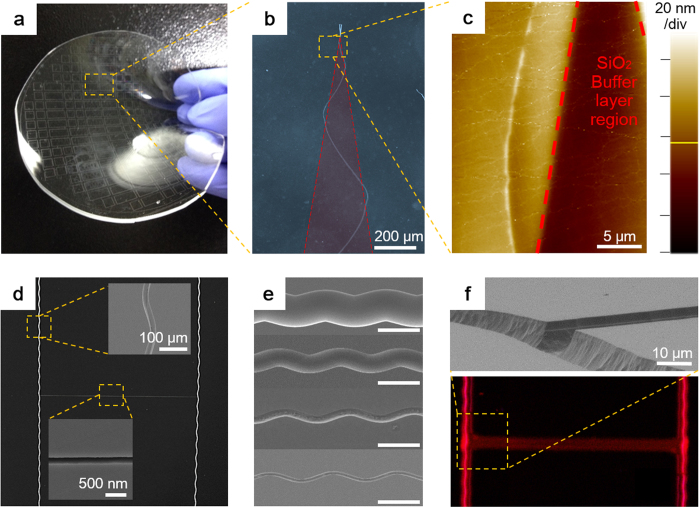
Large area nano/micro channel fabrication. (**a**) Waver scale nanocrack patterns transferred to polydimethylsiloxane (PDMS). (**b**) Optical microscopic image of inversed pattern of a nanocrack on PDMS. (**c**) Atomic force microscope (AFM) image of transferred nanocrack on PDMS. (**d**) Multi-scale H-shaped nano/micro channel fabricated by selective wet etching of existing cracks on (110) Si substrate. (**e**) SEM images of etched oscillatory cracks having various width. The inset scale bar is 100 μm. (**f**) SEM image of the intersection of widened channel by wet etching (top). Florescence image of quantum dot nanofluidic in the widened H-shaped channel fabricated from the controlled nanocrack (bottom).

There are various potential applications of the nanopatterns fabricated by precise manipulation of the oscillatory cracks. Besides the complex micropatterned letters with nanoscale width demonstrated in Fig. 2a, other fast and feasible application is to use the cracks as a nanochannel. Since the crack itself is naturally a channel structure with nanoscale width, it can easily be replicated by polydimethylsiloxane (PDMS) at wafer scale as shown in Fig. 6a,b. A magnified optical image of a specific area, shown in Fig. 6b, reveals a transferred crack pattern to PDMS. A three dimensional profile obtained via AFM (atomic force microscopy), shown in Fig. 6c, confirms that the embossed feature is the transferred crack pattern. Figure 6d shows a multi-dimension oscillating micro channel fabricated by subsequent wet etching of oscillatory cracks. Generally, adhesion between masking layer and substrate is important for microfluidic channel fabrication by wet etching, otherwise an additional deposition process is required. Usually, a metal layer on silicon substrate is patterned via lift-off process. Moreover, the minimum pattern resolution of this process is up to 2 μm using conventional aligner, thus creating features less than the minimum resolution is difficult especially when using isotropic wet etching. However, the Si_3_N_4_ film adheres to Si substrate solidly as a masking layer, less than 2 μm features can be made with simple conventional etchant: HNA (HF/Nitric/Acetic) solution as shown in Fig. 6d^26^. Strong adhesion of the Si_3_N_4_ film to Si substrate also facilitates repetitive wet etching without additional deposition or patterning. Figure 6e shows the result of multiple isotropic wet etching. The channel width can be expanded up to several hundreds of micrometers under 10 min total etching time.

The channels fabricated by wet etching of the cracks are directly applicable to nano/micro fluidics. As shown in Fig. 6f, smooth top surface of the substrate is advantageous for PDMS or glass to adhere well for closed channel fabrication. An expanded channel capped with a PDMS block is filled with quantum dot solution as shown in Fig. 6f. Unlike isotropic wet etching, use of anisotropic etchant makes a triangular channel section profile (see supplementary information Fig. 7s). The Si-to-Si_3_N_4_ etch selectivity of anisotropic etchant such as KOH or TMAH is much higher than that of HNA solution, in turn the Si_3_N_4_ thin film adjacent to crack opening does not collapse or fracture after etching. This is advantageous since the channel can be capped simply by bonding or spin coating. In general, fabrication of nano/microchannels equivalent to those demonstrated in this study in width and length will be even difficult with conventional lithography or electron beam lithography which is expensive, time consuming and not scalable to large size. Using manipulation of the cracks obviates such difficulties and dramatically simplify the process because cracks are self-generating in nature and can propagate all over the substrate and therefore easily scalable to very large size which is impossible with conventional processes.

## Conclusion

We have shown fabrication of various sophisticated nanopatterns by manipulating oscillatory cracks via Si_3_N_4_ film and SiO_2_ buffer layer. The oscillatory crack shows optical wave-like characteristics such as refraction, reflection, amplification, total internal reflection, and evanescent wave. These unique optical wave like characteristics of oscillatory crack are closely related to material and mechanical properties of the substrate and the film. Strategic modification of these mechanical and material properties allows elaborate control of the crack generation. Utilizing the crack and crack control, various sophisticated nanopatterns have been demonstrated at wafer scale. In addition, the nanopatterns fabricated in this fashion offer distinct advantages over conventional nano/microchannel fabrication methods in terms of simplicity, cost and scalability. We expect that our study can be used in many fields including nanofluidics and nanoelectronics which may require very long nanochannels with high consistency in LER and LWR.

## Methods

Silicon substrates with three different crystallographic orientations ((100), (110), and (111)) were cleaned by following standard wafer cleaning procedure. Notch-like structures were patterned on the substrates via photolithography followed by reactive ion etching (RIE). Subsequently, a 200 nm-thick silicon dioxide (SiO_2_) thin film layer was grown on the patterned substrate by thermal oxidation. The final SiO_2_ patterns were defined by photolithographic patterning followed by RIE. In order to control the thickness of SiO_2_ patterns, the substrates were submerged in a diluted HF solution (1:20). Various designs of the silicon dioxide film patterns were employed in the anticipated directions of crack propagation. As a last step, a stoichiometric silicon nitride (Si_3_N_4_) film was deposited on the substrates by chemical vapor deposition (CVD). The temperature and pressure condition of CVD were 800 °C and 200 mtorr, respectively. In order to induce high intrinsic stress on the silicon nitride film, dichlorosilane (H_2_SiCl_2_) and ammonia (NH_3_) at 30 cm^3^ min^−1^ (STP) and 100 cm^3^ min^−1^ (STP) were used respectively. The cracks self-propagate on the substrates during the CVD process. When the substrates were taken out, the final crack patterns were defined, thus no additional process is required. The substrates were then characterized and analyzed by scanning electron microscopy (SEM) and optical microscope.

## Additional Information

**How to cite this article**: Suh, Y. D. *et al*. Control and Manipulation of Nano Cracks Mimicking Optical Wave. *Sci. Rep*. **5**, 17292; doi: 10.1038/srep17292 (2015).

## Supplementary Material

Supplementary Information
