# Supplementary material for: Control and Manipulation of Nano Cracks Mimicking Optical Wave
Source: Sci Rep. 2015 Nov 27;5:17292. doi: 10.1038/srep17292 (PMC4661517; doi:10.1038/srep17292)
Supplement: Supplementary Information [file srep17292-s1.pdf]

Supporting Information

Control and Manipulation of Nano Crack Mimicking Optical Wave

By *Young D. Suh*[a], *Junyeob Yeo*[a, b], *Habeom Lee*[a], *Sukjoon Hong*[a], *Jinhyeong Kwon*[a], *Kyunkyu Kim*[a], *Seung Hwan Ko*[a]

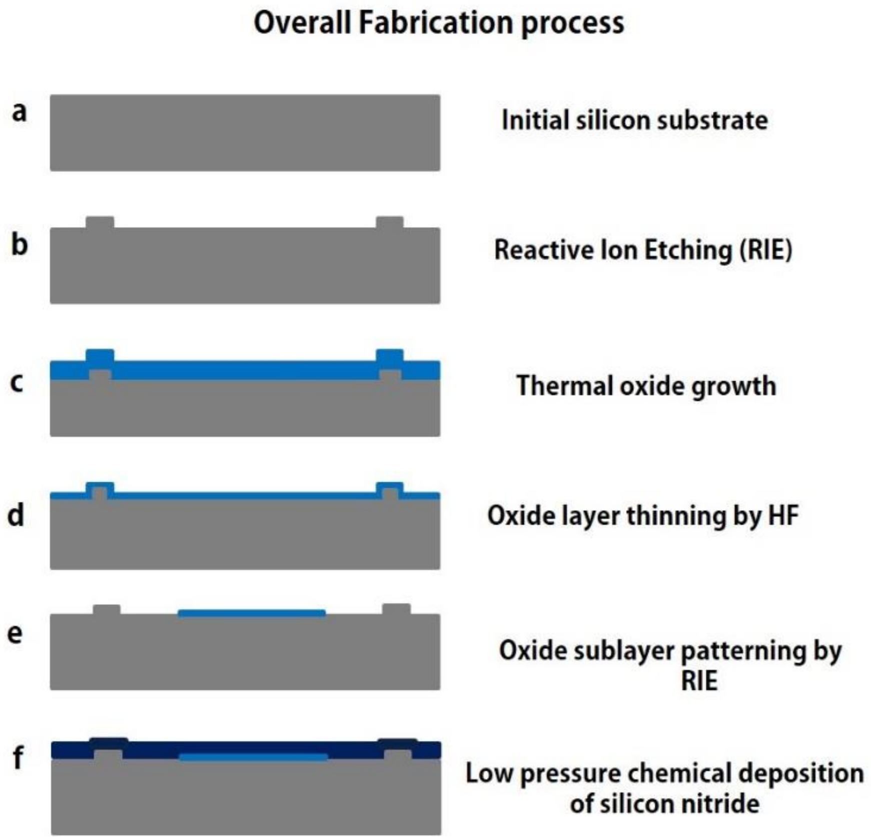

Figure 1S. Sequence for preparation of crack sample

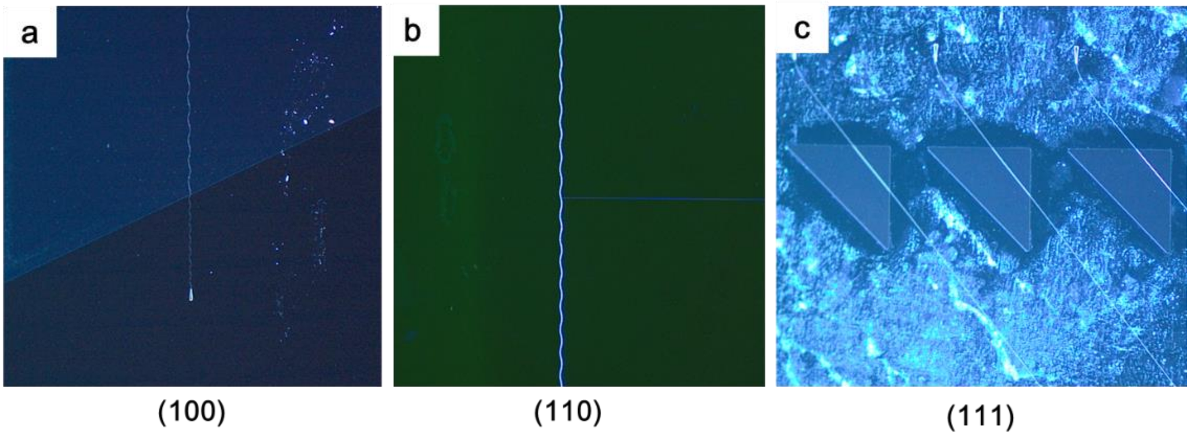

Figure 2S. Different crystalline structure silicon wafer. Structure silicon wafer with different crystal orientations have been tested for oscillatory crack formation. As shown in **Figure 2S**, no oscillatory cracks were found and SiO_2 buffer layer does not significantly alters the direction of the crack on substrates with different orientation, (110) and (111).

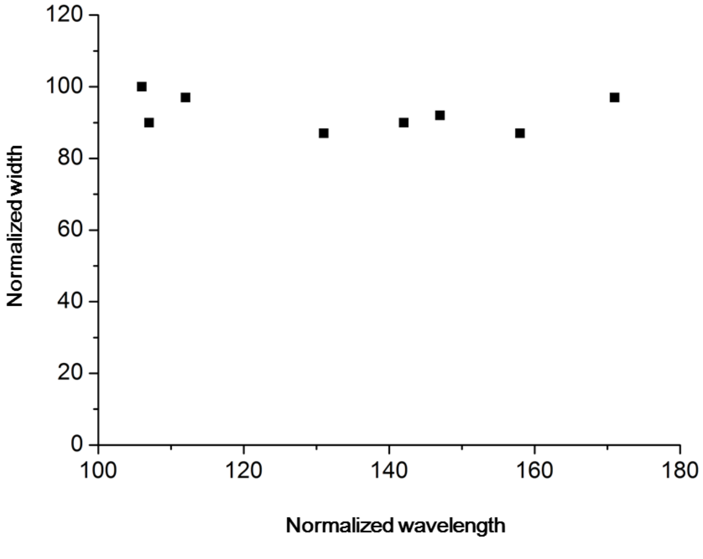

Figure 3S. Normalized crack width with various wavelength size. It appears that the width and the wavelength of oscillatory crack do not have correlation as shown in **Figure 3S**. The FIB section images in **Figure 4S** shows that there is no slippage between the films and the substrate, thus it is anticipated that the crack penetration into the substrate may be the governing factor for the width of the oscillatory cracks.

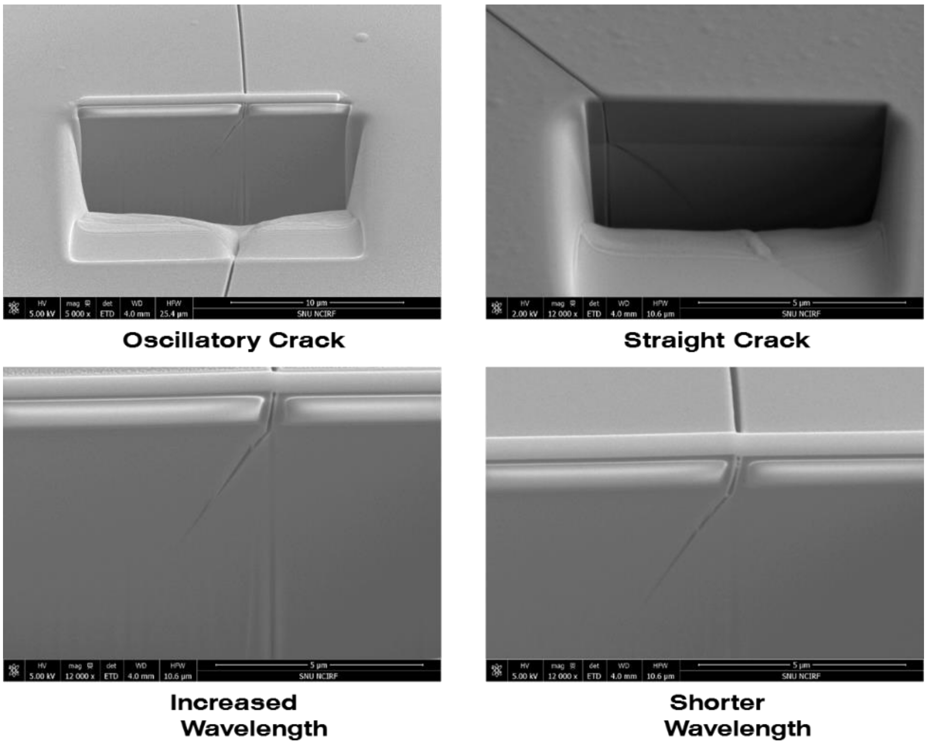

Figure 4S. Investigation of crack width with various types of crack. There is no significant difference in penetration depth and angle between the oscillatory and straight crack. The angle of penetration is approximately 55˚.

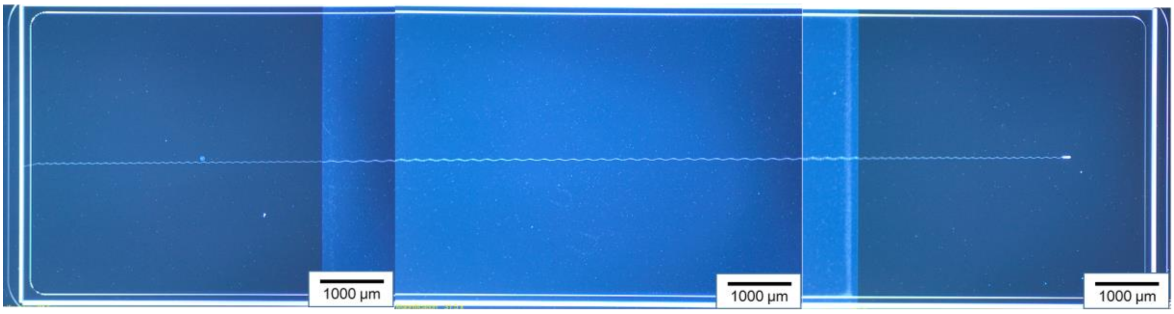

Figure 5S. Very large area patterning using oscillatory crack. The oscillatory crack propagates continuously unless there is another structure that creates sharp stress concentration. Using this property allows large area patterning using oscillatory crack. **Figure 5S** image was attained by joining three separate optical images. Even longer nanopatterns can be made if required. Middle section with different color indicates SiO_2 buffer layer where wavelength of the oscillatory crack has been elongated.

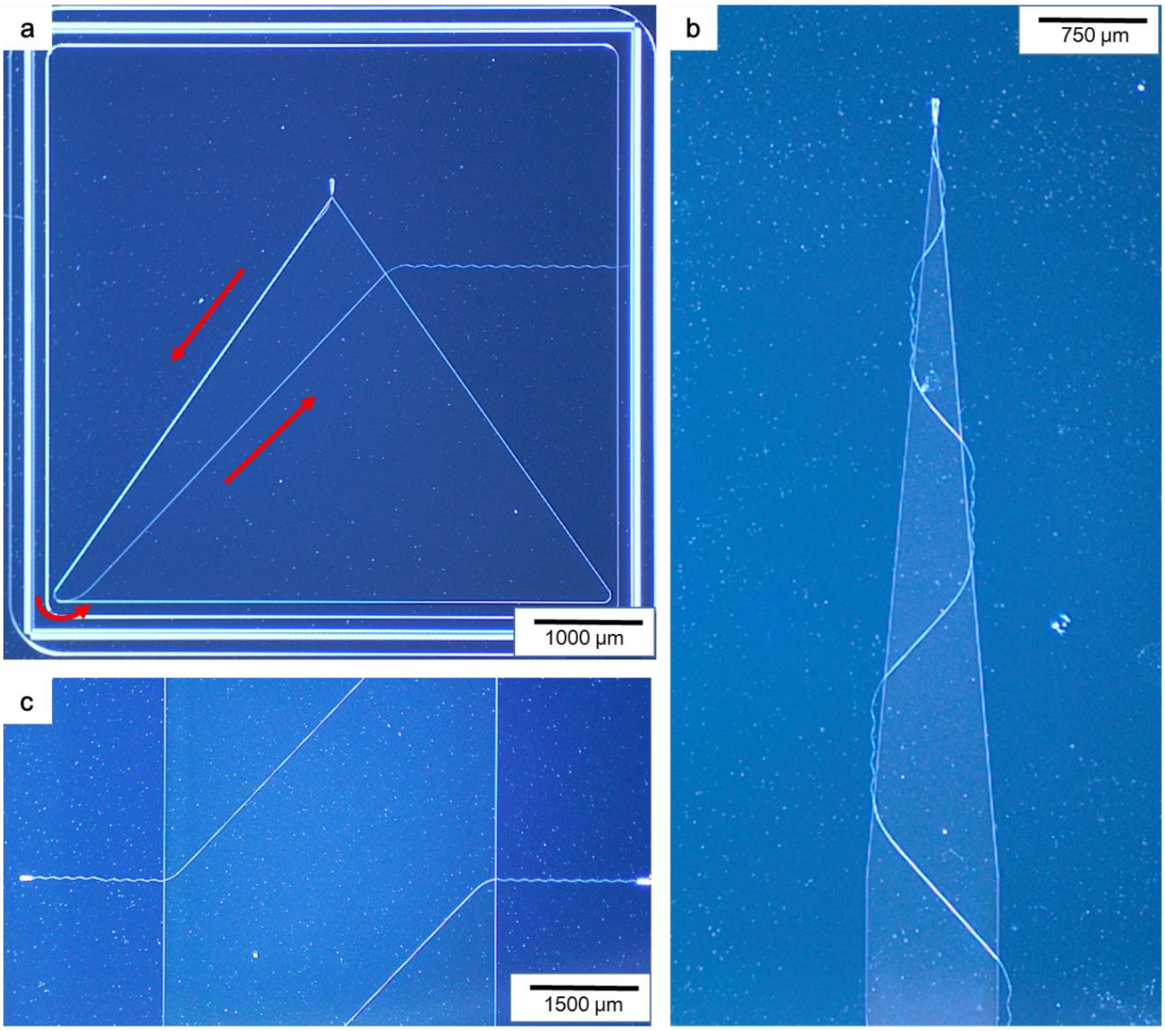

Figure 6S. Various area patterning using oscillatory crack. Using optical wave-like property of oscillatory crack, various nanopatterns can be fabricated. **Figure 6S** shows unique crack patterns attained using refraction of and combination of refraction and evanescent wave of the oscillatory crack.

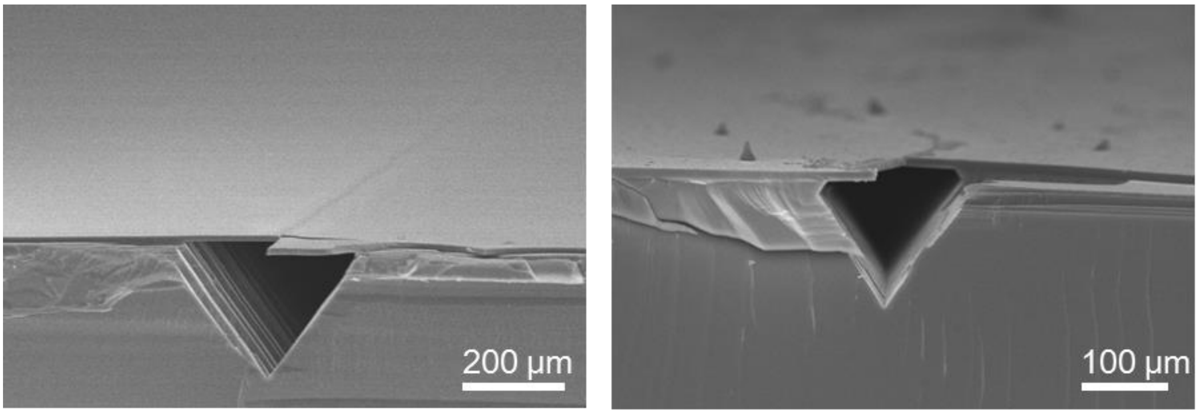

Figure 7S. Triangular channel section made by isotropic wet etching. Figure 7s shows channel section profile when wet etched by anisotropic etchant, KOH. Notice that the silicon nitride film is still intact, thus simple spin coating allows fabrication of completely closed channel.
